# Giant Penoscrotal Lymphedema—What to Do? Presentation of a Curative Treatment Algorithm

**DOI:** 10.3390/jcm12247586

**Published:** 2023-12-08

**Authors:** Denis Ehrl, Paul I. Heidekrueger, Riccardo E. Giunta, Nikolaus Wachtel

**Affiliations:** 1 Division of Hand, Plastic and Aesthetic Surgery, University Hospital, LMU Munich, 80336 Munich, Germany; 2 Centre of Plastic, Aesthetic, Hand and Reconstructive Surgery, University of Regensburg, 93053 Regensburg, Germany

**Keywords:** penoscrotal lymphedema, free vascularized lymph node transfer, lymphedema

## Abstract

Background: While rare, penoscrotal lymphedema (PL) is accompanied with devastating effects on the quality of life of patients. Moreover, especially for patients with excessive (giant) PL, no standardized curative treatment has been defined. This article therefore retrospectively evaluates the authors’ surgical treatment approach for giant PL, which includes resection alone or in combination with a free vascularized lymph node transfer (VLNT). Methods: A total of ten patients met the inclusion criteria. One patient dropped out of the study before therapy commenced. Eight of the nine remaining patients presented with end-stage (giant) PL. One patient presented with manifest pitting edema. All patients were treated with penoscrotal resection and reconstruction. Additionally, five patients received VLNT into the groin or scrotum. Results: The extent of the lymphedema was specified with a treatment-oriented classification system. The median follow-up was 49.0 months. No patient showed a recurrence. Patients who received VLNT into the scrotum displayed a significantly improved lymphatic transport of the scrotum. Conclusions: Advanced PL should be treated in a standardized surgical fashion as suggested by our proposed algorithm. VLNT from the lateral thoracic region into the scrotum must be considered. If treated correctly, surgical intervention of end-stage PL leads to good results with a low recurrence rate.

## 1. Introduction

While rare, penoscrotal lymphedema (PL) is typically endemic to Africa and Asia and is caused by lymphatic obstruction due to filariasis (typically Wuscheria bancrofti) or bacterial infection [1,2,3,4]. Other secondary causes include tumors, lymphadenectomy, radiotherapy, or disorders of the fluid balance, such as heart or kidney disease. Rarely, the edema can present as a symptom of a primary lymphatic malformations (i.e., Meigs and Milroy’s disease) [5,6,7]. Moreover, during the last two decades, an increasing number of articles have reported of cases with no clear etiology, termed idiopathic penoscrotal lymphedema (IPL) [5,8].

While benign, PL is accompanied with devastating effects on social life, sexual function, and hygiene [1,8,9]. First-line therapy for bacterial-associated PL is antibiotic treatment [10]. However, other etiologies regularly require surgery [5,8,11]. Here, primary closure, coverage with skin grafts, flap reconstruction, and microsurgical lymphaticovenous anastomoses (LVAs) in combination with surgical resection have been described [1,11,12,13]. Interestingly, while promising for other cases of lymphedema [8,14,15], vascularized lymph node transfer (VLNT) has not yet been assessed as a treatment for PL and was only described in two cases of PL with transplantation to the groin (17). Moreover, due to the rarity of the pathology to date, no clear consensus has been presented regarding diagnostics and, in particular, standardized treatment [4]. The purpose of this retrospective case series was therefore to evaluate the authors’ treatment approach for PL, which includes scrotal and penile resection and subsequent reconstruction alone or in combination with a free VLNT. Additionally, we set out to evaluate and present a new classification of PL and to define an algorithmic approach for the surgical treatment of PL. For this, we analyzed surgical and functional outcomes of scrotal and penile reconstructions without or with VLNT to suggest the best therapeutic option for PL.

## 2. Materials and Methods

### 2.1. Patient Selection/Inclusion Criteria

The clinical charts of all patients who presented with PL and received surgery were retrospectively reviewed from 2018 to 2022. The first patient reviewed presented with a gigantic IPL and therefore was published in 2018 [13]. Subsequently, patients with PL, both primary (idiopathic) and due to secondary causes, were transferred to our department on a regular basis. Indication for surgical therapy was an end-stage PL (stage III), according to the consensus statement of the International Lymphology Conference of 2020, or a failure of complete decongestive therapy [16]. Both primary (idiopathic) and secondary PL were included in this study. Any type of surgical procedure was included (scrotectomy/penile lymphedema reduction and/or VLNT). A chart review was performed to obtain a possible etiology, including serological and hematological laboratory tests to exclude parasite as well as bacterial infection. Moreover, charts were assessed for pre- and postoperative diagnostics, such as lymphoscintigraphy and/or MRI. Outcome parameters included postoperative complications, including surgical site infection, erectile dysfunction, donor site morbidity, and recurrence of lymphedema. The extent of recurrence was classified according to the consensus statement on the diagnosis and treatment of peripheral lymphedema of the International Lymphology Conference of 2020, where stage I (swelling and fluid accumulation) is classified as minor, stage II (manifest pitting edema) as moderate, and stage III (lymphostatic elephantiasis) as major recurrence [16]. Patients presenting with varicocele were not included in the study.

### 2.2. Scrotal and Penile Surgery

Scrotal resection (scrotectomy) and reconstruction was described in detail previously [1,3,13]. In accordance with our classification (Table 1), whenever the lymphedema affected the penis, resection of penile skin and subcutaneous tissue was performed. In case of mild to advanced PL, the penile skin is reconstructed using split skin grafts. For patients with advanced PL (buried penis), the penile skin is reconstructed with a dorsal flap from the mons pubis (wrap-around technique) [13]. In all patients, the excised tissue was sent for histological assessment.

### 2.3. Vascularized Lymph Node Transfer

For all patients with VLNT, CT angiography was performed preoperatively to assess donor as well as recipient vessels for transfer. The lymph nodes were harvested from the lateral thoracic region and axilla (anterior axillary lymph nodes/level 2). The lateral thoracic artery and vein were used as nutrient vessels. Additionally, a skin island was harvested to allow for adequate monitoring after microsurgical transfer. Initially, for patients 5 to 7, vascularized lymph nodes were transferred into the groin with arterial and venous anastomosis to the superficial circumflex iliac vessels, as described previously [17]. However, this approach does not correspond to the primary physiological lymph drainage from the scrotum. For this reason, we modified this approach in two patients (patient 8 and 9), such that vascularized lymph nodes were (corresponding to the primary physiological lymph drainage) transferred directly into the scrotum with the superficial external pudendal artery and vein as recipient vessels [18]. Microsurgical arterial and venous anastomoses were performed in standard fashion. Monitoring was performed with clinical and regular Doppler ultrasound control as well as using an Oxygen to See (O2C, LEA Medizintechnik, Gießen, Germany) device for continuous monitoring [19].

## 3. Results

**Figure 1:**
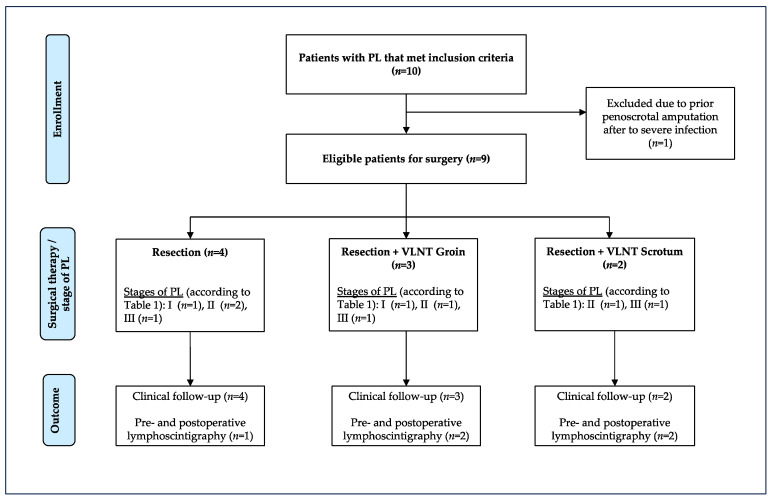
Study flow diagram. Nine patients were eligible for surgical therapy of penoscrotal lymphedema (PL). Eight of these patients presented with end-stage penoscrotal lymphedema (PL) (lymphostatic elephantiasis) and one patient presented with a manifest pitting edema [16]. The extent of the lymphedema was further specified with a treatment-oriented grading system (see also Table 1). All patients received scrotal and penile resection surgery in a single-stage procedure, with or without vascularized lymph node transfer (VLNT) into the groin or scrotum.

A total of ten patients met the inclusion criteria (Figure 1). The patients had a mean age of 41.7 years (24 to 59 years) at surgery. One patient dropped out of the study before surgical therapy commenced due to the fact that he presented with massive obesity (BMI of 52) and planned to lose weight and subsequently be treated with an abdominoplasty as well as penoscrotal reconstruction in combination with free VLNT. However, due to severe local infection, his scrotum and penis were amputated in another hospital.

Seven of the nine remaining patients presented with combined PL, and two patients presented with scrotal lymphedema only. Nearly all (eight out of nine) patients presented with stage III lymphedema (according to the consensus statement of the International Lymphology Conference of 2020, i.e., lymphostatic elephantiasis), while one patient presented with stage II lymphedema after failure of complete decongestive therapy, [16]. These all met the inclusion criteria for surgical therapy. As all patients were transferred to our department after extensive conservative treatment and/or with end-stage PL, no patient was treated with non-surgical options.

The extent of the lymphedema was further specified with a treatment-oriented classification system that was developed at our department (Table 1). An overview of the study cohort can be found in Table 2.

Seven patients presented with primary (idiopathic) and two with secondary PL (resection of the sigmoid colon due to diverticulitis (patient 3) and treatment for anal carcinoma, which included surgery and radiation therapy (patient 6)). All patients received scrotal and penile resection surgery in a single-stage procedure, with or without VLNT (Table 2).

The lymphedema of seven patients were preoperatively assessed with lymphoscintigraphy. One patient was assessed with an MRI scan, and one patient had no preoperative imaging of his PL. Preoperative diagnostic imagining for the eight patients showed none (three), minor (one), moderate (two), or severe (two) local impairment of scrotal lymphatic transport. One patient with moderate impairment of scrotal lymphatic transport also demonstrated a reduced lymphatic transport of the adjacent lower extremity (patient 6, secondary lymphedema after treatment for anal carcinoma).

The median follow-up was 49.0 months (16 to 67 months). All nine patients showed no recurrence in this period. However, one patient presented with a hydrocele testis during follow-up. He initially presented with a combined lymphedema of the scrotum and a buried penis (patient 6, also refer to Table 2) as well as lymphedema of both lower extremities after treatment for anal carcinoma and was initially treated with surgical resection of penile skin and subcutaneous tissue as well as VLNT to the groin.

Five patients (patients 2, 6, 7, 8, and 9) received postoperative lymphoscintigraphy. The postoperative lymphoscintigraphy of patients 2 and 7 demonstrated no significant change when compared to the preoperative findings (lymphoscintigraphy was taken five and four months after surgery, respectively). Postoperative lymphoscintigraphy (32 months after surgery) of patient 6 (scrotal and penile resection followed by VLNT into the groin) similarly showed no significant change in the scrotal lymph transport. Interestingly, a significant improvement in the lymphedema of the ipsilateral leg after VLNT into the groin was observed in this patient, while the lymphatic transport was unchanged in the contralateral leg.

Postoperative lymphoscintigraphy of patients who received VLNT into the scrotum (patients 8 and 9), however, showed significantly improved lymphatic transport of the scrotum three and four months after surgical intervention (scrotal and penile resection combined with VLNT into the scrotum).

For patients with vascularized lymph node transfer, no donor site morbidity on the trunk was observed. Additionally, no complications at the penoscrotal surgical site were observed. Histologic examination for all patients showed chronic lymphostasis, surrounded by fibrous tissue.

## 4. Discussion

**Figure 2:**
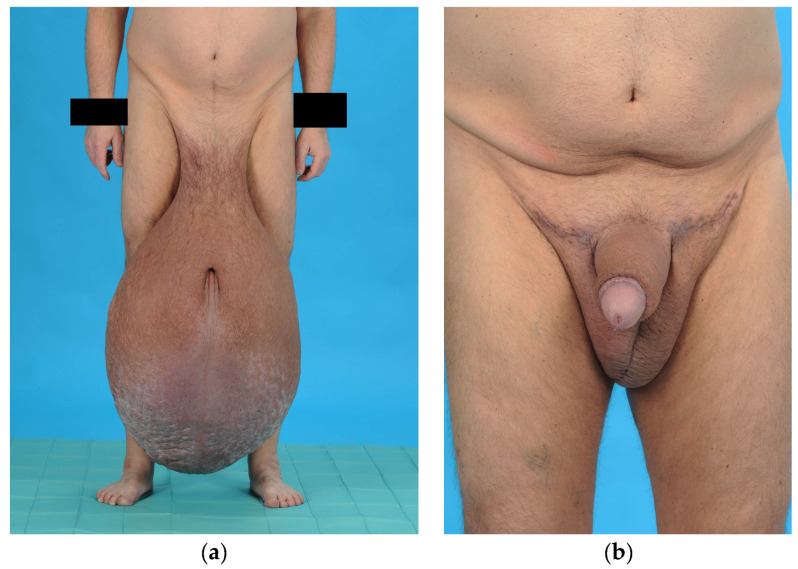
(**a**) Patient 1 with giant stage III penoscrotal lymphedema (PL), according to the classification in Table 1, before treatment and (**b**) 6 weeks after surgery (resection and flap reconstruction of scrotum and penis).

**Figure 3:**
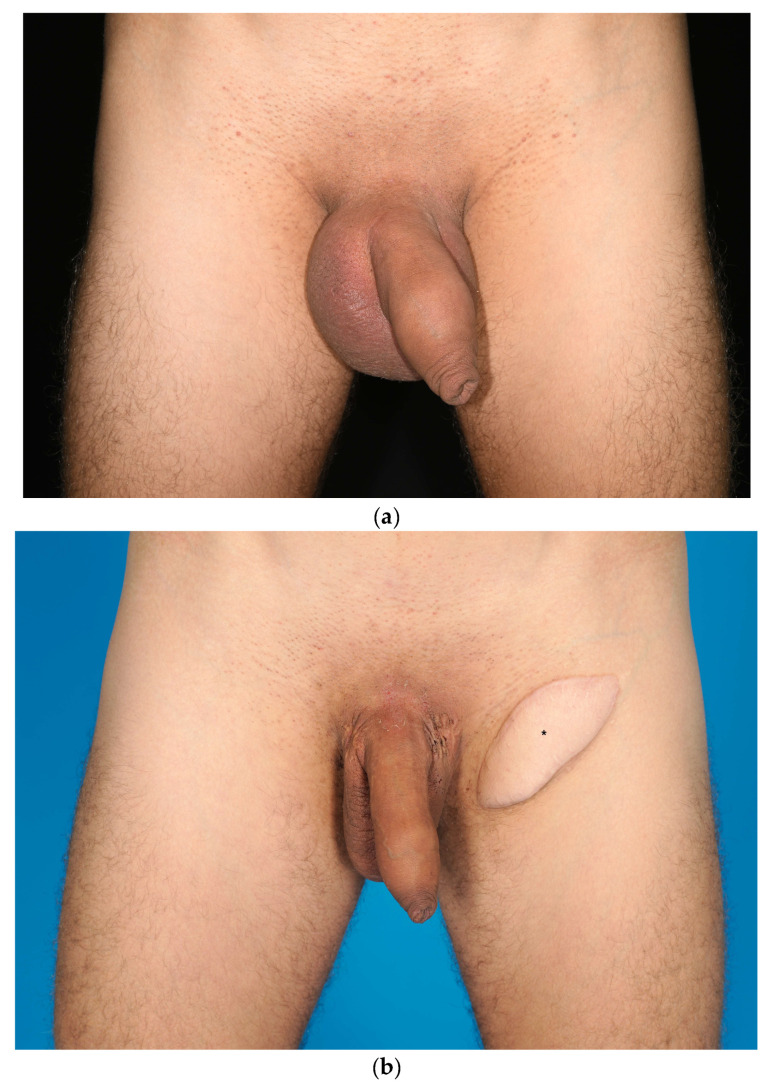
(**a**) Patient 7 with isolated lymphedema of the scrotum (stage I PL according to the classification in Table 1) before treatment and (**b**) 27 months after surgery (resection and flap reconstruction of scrotum combined with a vascularized lymph node transfer (VLNT) into the groin). The monitoring island of the VLNT in (**b**) is marked with an asterisk.

**Figure 4:**
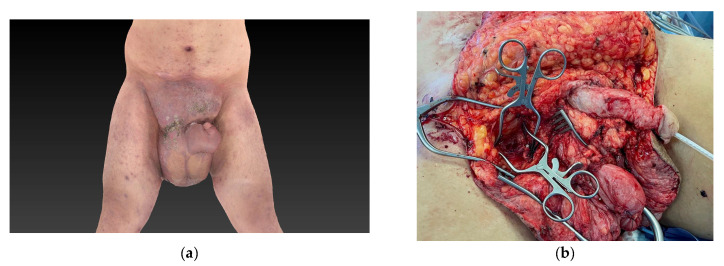

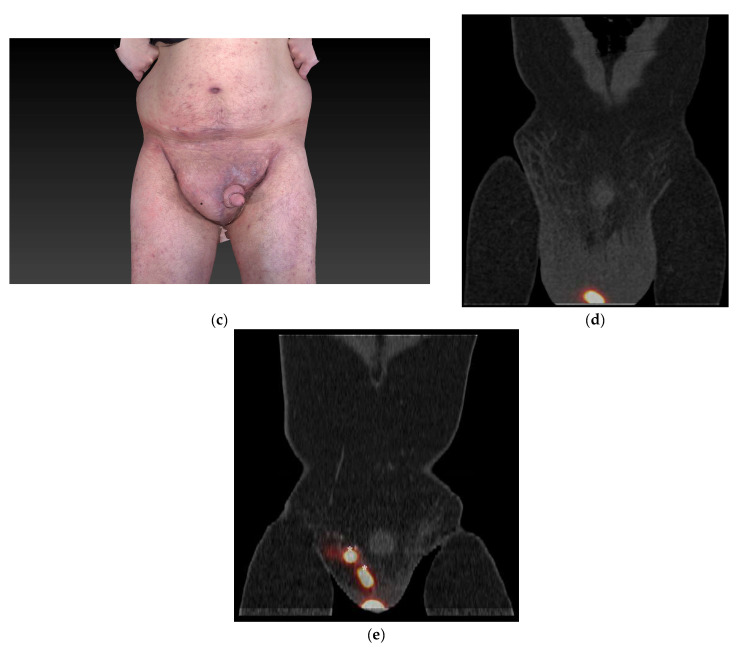
Images of advanced stage III penoscrotal lymphedema (PL), according to the classification in Table 1, (patient 8) (**a**) before treatment, (**b**) during surgery, and (**c**) after treatment. Panels (**d**,**e**) show low-dose SPECT-CT scans, before and after surgery. At initial presentation, the patient suffered from severe local infection and folliculitis and was therefore treated with long-term antibiotics (**a**). Surgery included resection and flap reconstruction of scrotum and penis combined with a vascularized lymph node transfer (VLNT) into the scrotum (**b**), with the superficial external pudendal artery and vein as recipient vessels (marked with an asterisk). Panel (**c**) shows the result 8 months after surgery. The monitoring island of the VLNT in (**c**) is marked with an asterisk. The skin and subcutaneous tissue of the island may be removed 6 months after surgery to further reduce the volume of the newly fabricated scrotum. Lymphoscintigraphy, including low-dose SPECT-CT scans, showed significantly improved lymphatic transport of the scrotum 3 months after surgical intervention (**e**), when compared to preoperative findings (**d**). The transplanted lymph nodes in (**e**) are marked with an asterisk.

Filarial infection with Wuscheria bancrofti, Brugia malayi or timori represents the most common etiology for lymphedema worldwide [4,5,20]. Consequently, most cases of PL are caused by this infection [4,5]. However, in regions where filarial worms are rarely found, such as Europe, Northern America, and Australia, other etiologies cause the majority of PL. Moreover, a growing number of idiopathic cases in these regions have been described previously [5,8,11]. Our study confirms these findings, as none of the patients showed filarial infection and only two presented with secondary PL. The other patients were classified as primary (idiopathic) PL (Table 2). Considering the excessive dimensions of the majority of lymphedema presented in this study and/or poor efficacy of previous long-term conservative treatment options, all patients received surgical treatment (Figure 2, Figure 3 and Figure 4) [4,8].

PL is a rare pathology, and a reduced number of patients is therefore to be expected [4]. Nevertheless, the present study encompasses one of the largest collections of clinical cases of advanced PL. While two other studies included more patients in total, the authors of both studies recruited a majority of patients with early-stage PL, where no or only minor surgical intervention (i.e., circumcision) was necessary [4,8]. Moreover, when assessing previous classifications of PL, we found these focus predominantly on etiologic or morphologic aspects [5,8]. In the current study, the extent of the PL was therefore further specified with a new classification (Table 1) [16]. Importantly, here, we grade PL according to the subsequent surgical procedure necessary for treatment.

Guiotto and colleagues thoroughly reviewed the previously published surgical treatment options for the therapy of PL [11]. The authors identified three surgical techniques, where two groups represent palliative treatment concepts: surgical resection and primary closure, or skin graft and resection followed by reconstruction with local flaps. A third group with curative intent was identified. Here, resection was followed by microsurgical LVA to improve lymphatic flow in the penoscrotal area. Interestingly, to date, only Phan et al. have reported two cases of VLNT to the groin for the treatment of PL [17]. This is in contrast with promising data on LVA and, in particular, VLNT for other advanced types of lymphedema [14,21,22]. Indeed, while LVA seems to be more effective in earlier stages of lymphedema, lymphatic vessels become sclerotic in more advanced stages, making VLNT the better option for advanced PL [11,14]. Therefore, while treating advanced PL, we introduced VLNT as therapeutic treatment for PL.

Anatomically, the first lymphatic drainage of the subcutaneous tissue and skin of the scrotum, as well as penis, is via the medial group of the superficial inguinal lymph nodes [11]. A previous publication describes lymph node transfer into the groin for the treatment of PL [17]. It is unclear, however, if a vascularized lymph node transfer into the groin only improves lymphatic drainage of the ipsilateral lower limb and if the combined resection of PL alone is responsible for the results observed by us and others. Moreover, it seems logical to transfer the lymph node into the anatomically affected area, i.e., the scrotum, thereby increasing the likelihood of improved lymphatic drainage. During our study, we therefore altered the VLNT from the groin to the scrotum.

**Figure 5:**
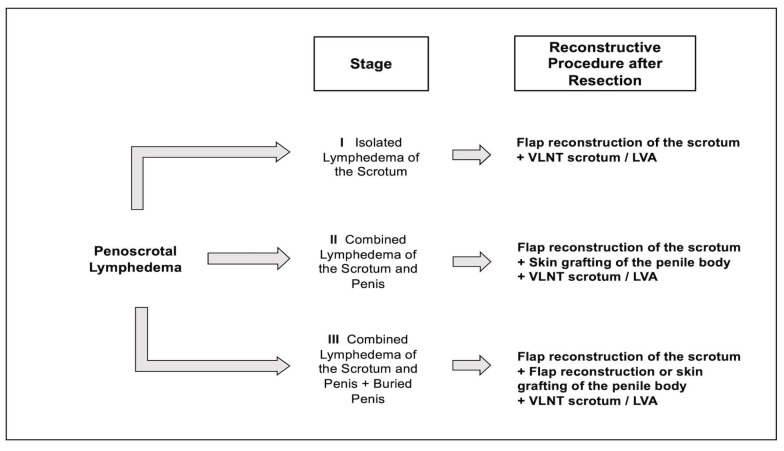
Proposed therapeutic algorithm for the treatment of penoscrotal lymphedema (PL) (according to the proposed treatment-oriented classification presented in Table 1). Prior to any surgical intervention, all conservative treatment options must have been pursued. If these fail, the following surgical options should be considered according to the stage of PL. For resection, standardized incisions should be performed as described previously by us and others [1,3,13]. Reconstruction of the scrotum can then be performed using flaps containing excess skin and subcutaneous tissue from the medial thigh (**stage I PL**). At **stage II PL**, reconstruction of the penile body is best accomplished with skin grafting. Due to the extensive skin excess around the mons pubis in **stage III PL**, sufficient healthy skin and subcutaneous tissue is available for flap reconstruction of the penile body in most cases. If flap reconstruction is not possible due to poor quality of tissue, we recommend skin grafting of the penile body (i.e., analogous to stage II PL). In combination with scrotal and penile reconstruction, we recommend a vascularized lymph node transfer (VLNT) directly into the anatomically affected area of the scrotum, thereby increasing the likelihood of improved lymphatic drainage. Alternatively, lymphaticovenous anastomoses (LVA) can be performed.

Our clinical experience as well as the findings of the current literature led to the development of an algorithm where we propose a therapeutic protocol with the intent for curative treatment of advanced PL (Figure 5). In our opinion, standardized resection, flap reconstruction, and/or skin grafting (for the penile body at stage II PL, also see Table 1) in combination with VLNT into the scrotum seems to be the best option for a curative treatment for advanced PL. Indeed, similar protocols have been previously proposed for the treatment of upper and lower extremity lymphedema with good long-term results [23,24,25]. These demonstrate the beneficial effect of a combined therapeutic approach of reductive and microsurgical procedures for the treatment of lymphedema. The resection of tissue reduces the increased solid tissue component (predominantly fibroadipose tissue as well as proinflammatory cytokines), leading to a decrease in overall lymphatic load. Microsurgical procedures, such as VLNT and LVA, effectively improve the lymphatic transport and thereby prevent a re-accumulation of lymphatic fluid in the affected region [23,25].

In the present study, no patient demonstrated with a recurrence, regardless of whether a VLNT was performed or not (Table 2). Nevertheless, the postoperative lymphoscintigraphy of patients with VLNT into the scrotum showed significantly improved lymphatic transport (patients 8 and 9), while the lymphoscintigraphy of patients who received no VLNT or a VLNT into the groin (patients 2, 6, and 7) was unchanged for the scrotum. Interestingly, patient 6 presented an improved lymphatic transport of the ipsilateral leg after receiving a VLNT into the groin. These findings therefore support our proposed treatment algorithm for PL, as they suggest an advantage of a VLNT into the scrotum, as opposed to no transfer or a transfer into the groin.

However, our conclusions remain suggestive as our study encompasses a limited number of cases as well as three different therapeutic approaches in an already small study population (resection alone and in combination with a VLNT into the groin or the scrotum). This is a clear limitation of our study. Moreover, our suggested algorithm is predominantly based on our clinical experience as well as similar protocols that were presented previously for other areas affected by chronic lymphedema (Figure 5) [23,25]. Thus, large-scale multicentric studies, with a prospective and randomized study design, are warranted. These would overcome the limitations caused by the low overall incidence of PL. A future study design should include a prospective assessment of the possible benefit of VLNT into the scrotum to effectively treat PL. Additionally, our study lacks objective outcome measurements for the quality of life as well as aesthetic aspects. While it seems highly plausible that these parameters are improved after treatment (Figure 2 and Figure 4), the lack of an objective assessment of these is a limitation of this study.

## 5. Conclusions

End-stage PL, although rare, should be treated in a standardized surgical fashion. VLNT from the lateral thoracic region into the scrotum must be considered as the donor-site morbidity is low and it seems highly likely that this procedure offers a curative treatment option. If treated correctly, surgical intervention of end-stage PL (lymphostatic elephantiasis) results in good outcomes with a low recurrence rate (Figure 2 and Figure 4). Moreover, moderate PL with pitting edema may also be successfully treated with the proposed surgical technique (Figure 3).

## Figures and Tables

**Table 1:** Treatment-oriented classification of penoscrotal lymphedema.

Stage	Criteria	Number of Patients
I	Isolated Lymphedema of the Scrotum	2
II	Combined Lymphedema of the Scrotum and Penis	4
III	Combined Lymphedema of the Scrotum and Penis and Buried Penis	3

**Table 2:** Overview of the surgical procedures performed in the study cohort. Eight patients presented with end-stage penoscrotal lymphedema (PL) (lymphostatic elephantiasis), and patient 7 presented with a manifest pitting edema [16]. The extent of the lymphedema was further specified with a treatment-oriented grading system (Table 1). For all patients, scrotal and penile resection and subsequent reconstruction alone, combined with vascularized lymph node transfer into the groin (VLNT Groin), or combined with vascularized lymph node transfer into the scrotum (VLNT Scrotum) was performed. All patients were treated in a single-stage procedure.

Patient	Etiology	Treatment-Oriented Stage of PL	Surgical Procedure	Follow-Up Period (Months)
1	Primary	III	Resection	67
2	Primary	I	Resection	59
3	Secondary	II	Resection	52
4	Primary	II	Resection	45
5	Primary	II	Resection + VLNT Groin	58
6	Secondary	III	Resection + VLNT Groin	49
7	Primary	I	Resection + VLNT Groin	37
8	Primary	III	Resection + VLNT Scrotum	18
9	Primary	II	Resection + VLNT Scrotum	16

## Data Availability

The data presented in this study are available on request from the corresponding author. The data are not publicly available due to privacy concerns of the patients enrolled in this study.
